# A Facile and Low-Cost Route to Heteroatom Doped Porous Carbon Derived from Broussonetia Papyrifera Bark with Excellent Supercapacitance and CO_2_ Capture Performance

**DOI:** 10.1038/srep22646

**Published:** 2016-03-03

**Authors:** Tongye Wei, Qi Zhang, Xiaolin Wei, Yong Gao, Huaming Li

**Affiliations:** 1 Hunan Key Laboratory of Micro-Nano Energy Materials and Devices, Department of Physics, Xiangtan University, Xiangtan 411105, Hunan Province, P. R. China; 2 College of Chemistry, Xiangtan University, Xiangtan 411105, Hunan Province, P. R. China

## Abstract

In this work, we present a facile and low-cost approach to synthesize heteroatom doped porous carbon via hydrothermal treatment of stem bark of broussonetia papyrifera (BP) as the biomass precursor in diluted sulfuric acid, and following thermal activation by KOH at 800 °C. The morphology, structure and textural property of the prepared porous carbon (PC) are investigated by scanning electron microscopy, transmission electron microscopy, N_2_ sorption isotherms, and X-ray photoelectron spectroscopy. The porous carbon possesses a high BET surface area of 1759 m^2^ g^−1^ and an average pore size of 3.11 nm as well as hetero-oxygen (9.09%) and nitrogen (1.7%) doping. Such porous carbon shows outstanding capacitive performances of 416 F g^−1^ and 300 F g^−1^ in three and two-electrode systems, respectively. As a solid-state adsorbent, the obtained porous carbon has an excellent CO_2_ adsorption capacity at ambient pressures of up to 6.71 and 4.45 mmol g^−1^ at 0 and 25 °C, respectively. The results present one novel precursor-synthesis route for facile large-scale production of high performance porous carbon for a variety of great applications including energy storage and CO_2_ capture.

Activated carbons (ACs) have been attracting a lot of investigation in previous decades. The reason for this attention is due to it is cheap, abundant, environmentally safe, commercially available and a sustainable biological resource^1^. Generally, ACs are usually prepared by a two-step process, in which carbon precursors are pyrolyzed at temperatures in the range of 600 ~ 900 °C in an inert atmosphere such as Ar or N_2_, followed by physical activation at elevated temperature in the presence of suitable oxidizing atmosphere chemical activation process using chemical agents such as KOH, NaOH, H_3_PO_4_, and ZnCl_2_^1^,^2^,^3^,^4^. It has been demonstrated that the incorporation of heteroatoms, such as sulfur, boron, nitrogen, and oxygen, into the carbon lattice can significantly enhance mechanical, semi-conducting, field-emission, and electrical properties of carbon materials. For example, nitrogen doping is considered to be the most promising method for enhancing surface polarity, electric conductivity and electron-donor tendency of the ACs, thereby improving their performances in CO_2_ capture, electric double-layer capacitors, fuel cells, and catalysis^5^. To prepare these materials, one approach is to use nitrogen-containing original precursors^6^ or ionic liquids^7^ for pyrolysis, and another approach is to post-treat carbon with N-containing dopants such as ammonia, amine, or urea^8^. Biomass is an attractive original precursor, which is cheap, environment friendly and readily available in high quality and quantity. In addition to its carbon and water content, biomass is rich in elements such as sulfur, nitrogen, and phosphorus, as well as metals such as Fe, Cu, and others^9^. Up to now, however, only a small portion of biomass materials such as pig bones, egg white protein, amaranth, hemp^10^, bean dregs^11^,^12^, seaweeds^13^ or seaweed biopolymers^14^,^15^, etc., are used as carbon precursors for supercapacitor electrodes and CO_2_ capture. Therefore, the search for new precursors, which are cheap, accessible and with potential to create significant economic valorisation, is highly desired. Herein we employ stem bark with complex multilayered structure from broussonetia papyrifera (BP) as the carbon precursor to develop a simple and facile method for the large-scale production of nitrogen doped ACs.

It is well known that BP is a species of flowering plant in the family moraceae. Importantly, the initial oxygen and nitrogen contents of the stem bark from BP are around 50 wt% and 1.5 wt%, respectively^16^,^17^,^18^,^19^. Therefore, the BP stem bark should be a promising carbon precursor for the production of heteroatom-doped ACs due to its cheap, abundant, and a sustainable biological resource. In the present study, we prepared hetero-oxygen (9.09%) and -nitrogen (1.7%) co-doped porous carbon with a high BET surface area via hydrothermal treatment of stem bark with complex multilayered structure from BP as the biomass precursor, and following thermal activation by KOH at 800 °C. The applications of this novel heteroatom doped porous carbon in high-power supercapacitors and CO_2_ capture are also demonstrated.

## Results and Discussion

### Synthesis and Characterization of Nitrogen Doped Porous Carbon

**Figure 1:**
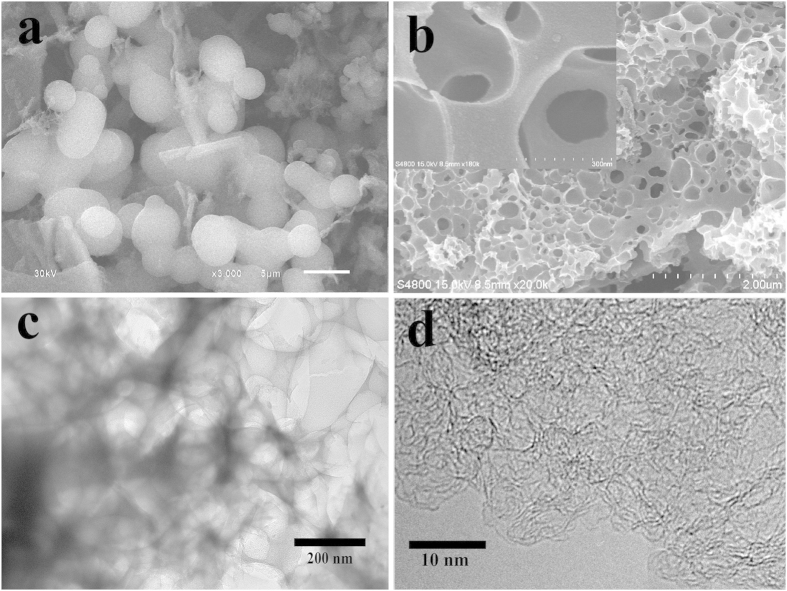
SEM images of biochar (**a**) and the PC-800 (**b**). (**c,d**) TEM images of the PC-800 at different magnifications.

As mentioned previously, heteroatom doped porous carbon with high BET surface area was prepared via a two-step progress, in which the BP stem bark was first hydrothermal treated in 1 M diluted sulfuric acid and then thermal activated by KOH at 700~900 °C. The resultant carbon materials are denoted as PC-700, PC-800, and PC–900, respectively. In the first step of hydrothermal treatment, the BP stem barks undergo acid-catalyzed dehydration, fragmentation, rearrangement and polymerization reactions to produce acid-insoluble intermediate biochar including spherical conjugated aromatic carbonaceous materials^20^,^21^,^22^ and pseudo-lignin, which consists of carbonyl, carboxylic, aromatic and aliphatic structures. Usually, spherical structure with diameter of 2~5 μm and flake from residual BP stem bark can be observed, which is consistent with our SEM observations (see Fig. 1a). Then during thermal activation at high temperature, the KOH activation involves primary reaction of 6KOH + 2 C = 2 K + 3 H_2_ + 2 K_2_CO_3_, decomposition of K_2_CO_3_ to K_2_O, and reactions of K/K_2_O/K_2_CO_3_/CO_2_ with carbon^23^,^24^. In addition, the abundant oxygen functional groups in biochar can react with KOH during heating, and generate H_2_O and CO_2_ gases because of dehydration and decarboxylation^24^. Particularly, upon heating to high temperature, the biochar undergo much stronger dehydrogenation and intermolecular dehydration reactions. As a result, serious structural changes are induced, leading to a much more porous network in final products. Considering the fact that the synthesis parameters, such as the hydrothermal temperature, H_2_SO_4_ concentration, biochar/KOH mass ratio, and activation temperature, play important roles in the regulation of the capacitance performance of the final porous carbons, a series of experiments were performed in order to screen the optimal synthesis conditions. Our experimental results confirm that the optimal synthesis conditions are: hydrothermal temperature = 170 °C, H_2_SO_4_ concentration = 1 M, biochar/KOH mass ratio = 1/2, and activation temperature = 700 ~ 900 °C (see Supporting Information, Figs S2 and S3 and Tables S1 and S2). Figure 1b shows the SEM image of the resultant PC–800, demonstrating that the prepared porous carbon sample possesses a continuous branched porous framework consisted of carbon nanosheets and fully interconnected pores ranges from several to hundreds of nanometers (Fig. 1b and the inset). The porous sheet-like structure is further studied by TEM observation. The typical TEM image (see Fig. 1c) reveals that the PC–800 has obvious porous texture. In addition, the localized graphitic structure which leads to an enhanced conductivity, can be seen from high-resolution TEM image in Fig. 1d.

**Figure 2:**
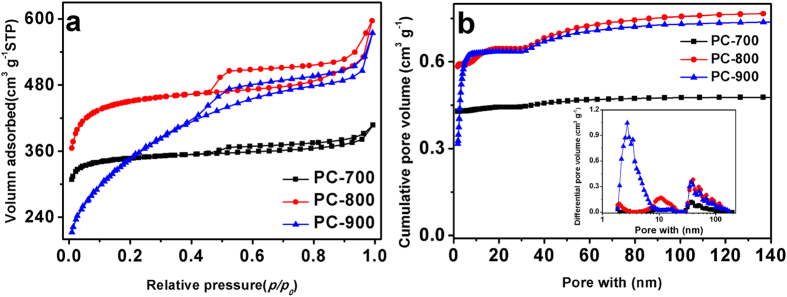
N_2_ adsorption-desorption isotherms (**a**) and pore size distributions (**b**) of the PC-700, PC-800, and PC-900.

The textural structure of the obtained porous carbons is investigated by N_2_ adsorption at 77.3 K. As shown in Fig. 2a, the isotherms of PCs exhibit a typical type-IV curve with an obvious type-H4 hysteresis loop at *p/p*_*o*_ = 0.45. The existence of type-H4 hysteresis loop is indicative of the presence of large mesopores embedded in a matrix with interconnected channels of much smaller size^25^,^26^. Figure 2b shows the pore size distribution (PSD) of the samples at different activation temperatures calculated according to NLDFT model. The meso and macro-pores increase with the temperature increment. The textural and electrochemical properties of these samples are summarized in Table 1 in detail. The PC–800 exhibits the BET surface of 1759 m^2^ g^−1^, which is much higher than that of PC–700 (1393 m^2^ g^−1^) and PC–900 (1229 m^2^ g^−1^) as well as the highest pore volume of 0.92 m^3^ g^−1^.

**Figure 3:**
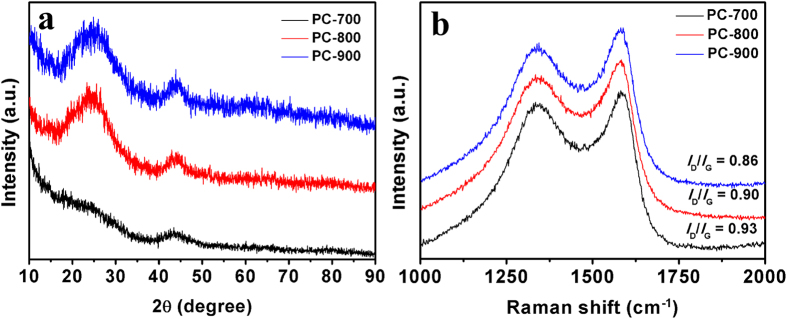
XRD patterns (**a**) and Raman spectra (**b**) of the PC-700, PC-800 and PC-900.

The XRD patterns of the obtained PC samples at 700, 800 and 900 °C are shown in Fig. 3a. All samples show two weak diffraction peaks centered at 2θ = 23.2° and 43°, respectively. The value of *d*_002_ is about 0.38 nm, larger than that of graphite (0.335 nm), implying a random combination of graphitic and turbostratic stacking^27^,^28^. In addition, with increasing calcination temperature, the intensities of the 2θ peaks at approximately 43 °C increases, due to the formation of higher degree of graphitic structure at higher carbonization temperature, which will greatly improve the electrical conductivity^29^,^30^,^31^. Figure 3b is the corresponding Raman spectra, exhibiting two bands at around 1339 cm^−1^ (D band) and 1582 cm^−1^ (G band) for all samples. The D band is related to the presence of disordered carbon structures, while the G band is associated with the vibration of sp^2^-hybridized carbon atoms in a graphitic layer^32^,^33^. The intensity ratio of D and G bands (*I*_D_/*I*_G_) is known to be an indicator of the degree of graphitization, and the smaller the *I*_D_/*I*_G_ ratio, the higher the degree of graphitization. Here, the *I*_D_ / *I*_G_ peak ratio of PC–700, PC–800, and PC–900 are determined to be 0.93, 0.90, and 0.86, respectively. The results are consistent with those from XRD patterns.

**Figure 4:**
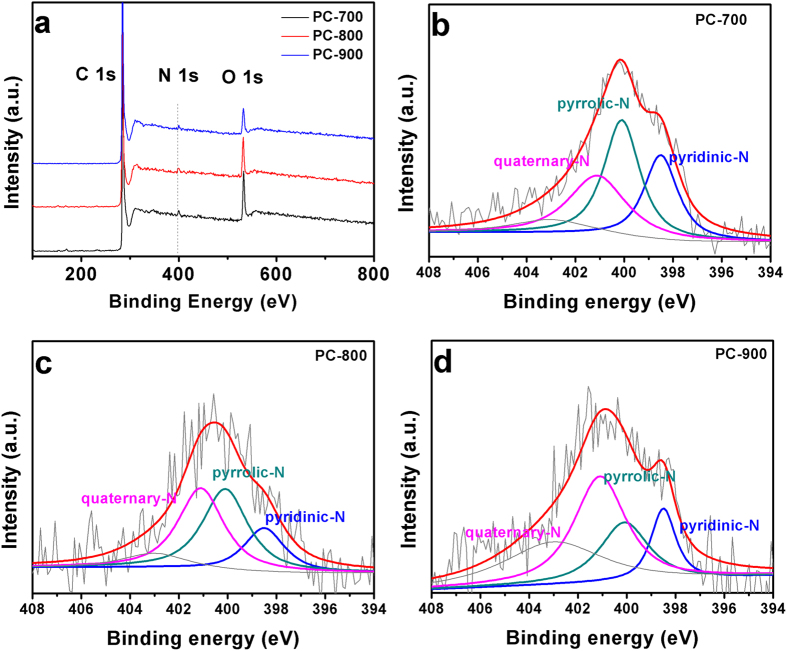
XPS survey spectra (**a**) and High-resolution XPS spectrum of N1s peaks (**b–d**) of the PC-700, PC-800, and PC-900.

XPS measurements were further used to analyze the components and chemical bonding at the surface of the BP-derived porous carbons. As shown in Fig. 4a, all samples exhibit a predominant C 1 s peak at 285 eV, a weak N 1 s peak at ca. 401 eV, and an O 1 s peak at ca. 533 eV. Table S3 provides the C, O, and N contents obtained from XPS analysis. The N contents are found to be 1.66, 1.43, and 0.99 atom% for the PC-700, PC-800 and PC-900, respectively, while the O contents are 10.91, 9.09, and 6.42 atom%. Clearly, the N and O contents of the prepared samples are dependent on the activation temperature. The high resolution XPS spectra of N 1 s for PC–700, PC–800 and PC–900 (see Fig. 4b–d) reveal the presence of four types of N configurations. The peaks at 398.5, 400.1, and 401.1 eV indicate the N doping in the form of pyridinic-N, pyrrolic-N, and quaternary-N^30^,^34^, respectively. As reported previously, electrochemical performance of carbons can be further enhanced by surface functionalities of O and N) due to additional pseudocapacitance^35^,^36^,^37^.

### Capacitance of the BP-Derived PCs

**Figure 5:**
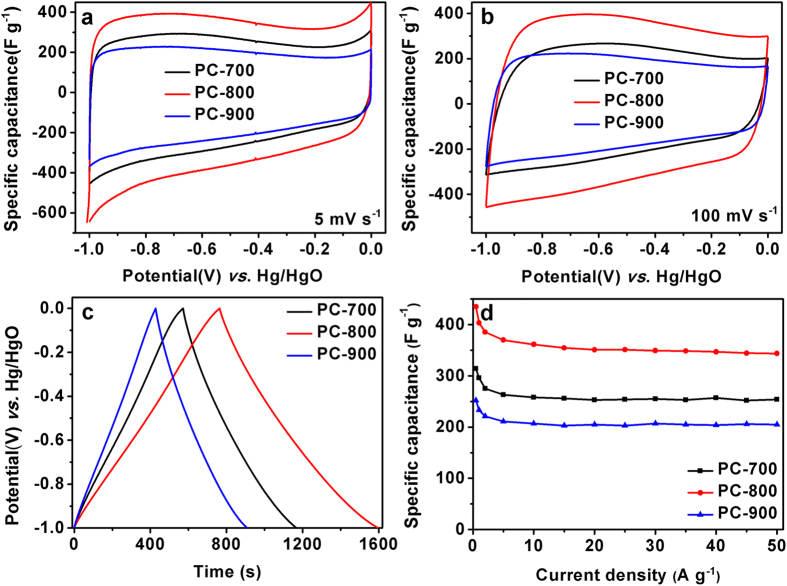
CV curves tested at a scan rate of 5 mV s^−1^ (**a**) and 100 mV s^−1^ (**b**), the GC curves tested at a current density of 0.5 A g^−1^ (**c**), the correlation of specific capacitances with current densities (**d**) of the PC-700, PC-800, and PC-900.

**Figure 6:**
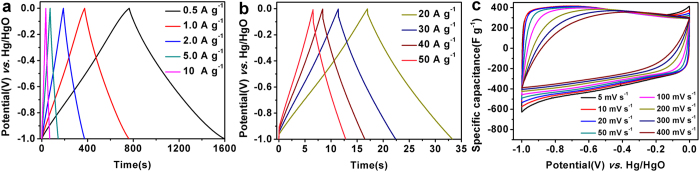
GC curves tested at 0.5–20 A g^−1^ (**a,b**) and the CV curves tested at scan rates of 5–400 mV s^−1^ (**c**) of the PC-800 electrode.

**Figure 7:**
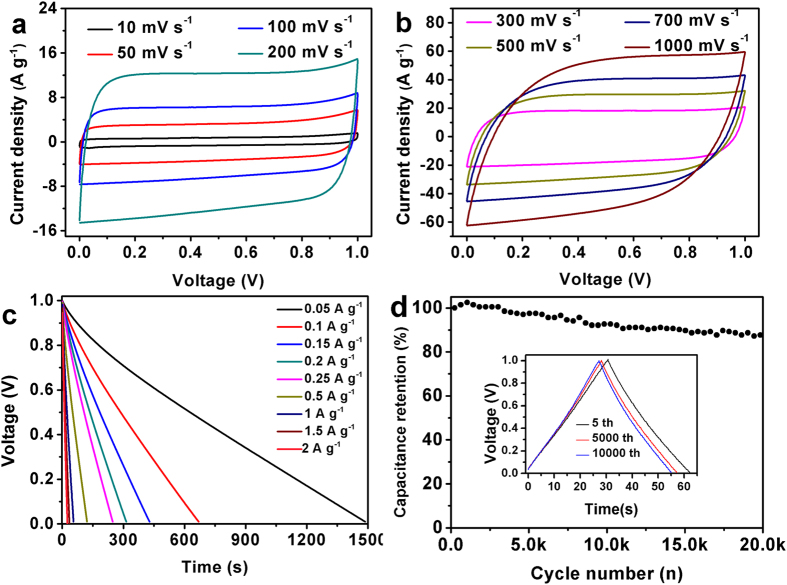
CV curves at 10–1000 mV s^−1^ (**a,b**), GC discharge curves of 0.05–2 A g^−1^ (**c**) and cycling performance (**d**) of the PC-800 based supercapacitor.

Electrochemical performance of BP-derived PCs as electrode materials for supercapacitors was initially estimated using a standard three-electrode system in 6 M KOH solution. The typical CV curves of the BP-derived PCs at a scan rate of 5 mV s^−1^ and 100 mV s^−1^ in the potential range between −1 and 0 V (*vs*. Hg/HgO) are shown in Fig. 5a,b. No matter at a lower scan rate of 5 mV s^−1^ (see Fig. 5a) or a higher scan rate of 100 mV s^−1^(see Fig. 5b) all PCs electrodes show a quasi-rectangular shape in the CV curves, suggesting an ideal electric double-layer capacitance behavior based on ionic adsorption and exchange. On the other hand, at a higher scan rate of 100 mV s^−1^, no dramatic change of the CV curves is observed. The GCD curves of these PCs electrodes are linear and display an isosceles triangle shape (Fig. 5c), indicating that the BP-derived PCs in KOH solution demonstrate ideal charge and discharge characteristics for electric double-layer capacitor. Figure 5d summarizes the gravimetric specific capacitance of three samples according to the GCD testing. It is found that the PC–800 electrode has the highest capacitance at the same current density. For instance, it possesses a specific capacitance of about 416 F g^−1^ at 0.5 A g^−1^, which is higher than those from the PC–700 and PC–900 electrodes. Moreover, the capacitance of the PC–800 electrode shows only a slight decrease to 333 F g^−1^ when the current density increased to 50 A g^−1^, suggesting good rate capability. Notably, the specific capacitance value of 416 F g^−1^, is higher than the reported values in aqueous electrolyte^38^,^39^. In addition, the PC–800 electrode shows better supercapacitor performance compared to electrodes made from various other carbon-based materials using other biomass as reported in the literatures^38^,^40^,^41^. The superior specific capacitance of the PC–800 electrode is ascribed to its high specific surface area, hierarchical porous structures, good conductivity, and heteroatom-doping. Considering that the rate capability is an important factor for the use of supercapacitors in power applications. The CV tests at different scan rates and GCD tests at different current densities were performed. As shown in Fig. 6c, with the scan rate increasing from 5 to 400 mV s^−1^, the CV curves of the PC–800 electrode become somewhat distorted but still retain a rectangular-like shape, suggesting an excellent high-rate capacitive behavior. Similarly, the GCD curves of the PC-800 electrode display a nearly isosceles triangle shape at different current densities (see Fig. 6a,b). More importantly, the GCD curves still maintain triangle shapes at a high current density of 50 A g^−1^, implying good coulombic efficiency (93%) and ideal capacitive behavior. The rate capability and cycling performance of the BP-derived PC electrodes based supercapacitor were further tested with a two-electrode system. The CV tested at different scan rates of 10 ~ 1000 mV s^−1^ and GCD tested at 0.05~2 A g^−1^ were utilized to examine the rate capability of the PC–800 based supercapacitor. As shown in Fig. 7a,b, the CV curves possess a typical rectangular shape at relative low scan rates. With increasing scan rate up to 1000 mV s^−1^, the CV curve still exhibits rectangular shape with only a little distortion, indicating an ideal electrochemical capacitive behavior with rapid diffusion and easy transportation of electrolyte ions to the interface of the electrode^42^. Figure 7c shows the GCD curves of the PC-800 supercapacitor. Clearly, the curve of 2 A g^−1^ still maintains linear and no obvious IR drop appears, suggesting characteristics of a little internal resistance and high rate capability of the PC–800 based supercapacitor. The cycling stability of the PCs based supercapacitor during the charge-discharge process is considered as another crucial factor in practical applications. As shown in Fig. 7d, the PC–800 based supercapacitor exhibits high capacitance retention of about 87.2% after 20000 charge-discharge cycles in a two-electrode configuration at 2 A g^−1^, indicating excellent electrochemical cycling stability.

### CO_2_ adsorption properties of the BP-Derived PCs

**Figure 8:**
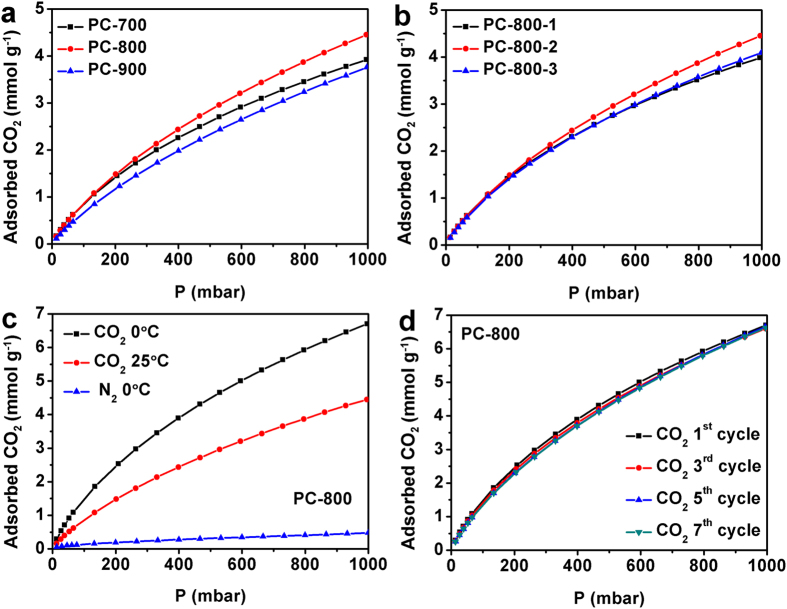
CO_2_ adsorption isotherms (**a,b**) on different porous carbons at 25 °C and 0–1000 mbar, (**c**) CO_2_ absorption isotherms and N_2_ adsorption isotherms on the PC-800 and (**d**) CO_2_ adsorption isotherms on the PC-800 at 0 °C and 0–1000 mbar within seven repeated cycles with regeneration.

The CO_2_ adsorption on BP-Derived PCs was investigated at 25 and 0 °C under atmospheric pressure (1000 mbar). The CO_2_ adsorption isotherms measured at ambient conditions for all BP-Derived PCs are presented in Fig. 8. The CO_2_ adsorption capacities for the PC-700, PC-800 and PC-900 are about 1.06, 1.08 and 0.85 mmolg^-1^ at 100 mbar, respectively (Fig. 8a). The results suggest that at very low pressure, the CO_2_ adsorption capacity of the porous carbons are highly depend on their nitrogen content (1.61%, 1.43% and 0.99% shown in Table S3)^11^,^19^. When the pressure increases to 1000 mbar, the CO_2_ adsorption capacities increase to 3.91, 4.45 and 3.76 mmol g^-1^, respectively, for the PC-700, PC-800 and PC-900, which is consistent with the BET results in Table 1. Particularly, the PC-800 has the unprecedented CO_2_ adsorption capacities of 4.45 and 6.71 mmol g^−1^ at 25 and 0 °C, respectively, which are higher than those of the reported carbons and comparable to the nitrogen containing carbons^19^,^31^,^43^. For example, the activated polypyrrole showed the CO_2_ uptake only of 6.2 mmol g^−1^ at 0 °C^44^,^45^ and active poplar anthers had the CO_2_ uptake only of 4.18 mmol g^−1^ at 25 °C^19^. It should be pointed that the N_2_ adsorption capacities for the PC–800 under ambient pressure at 0 °C is only 0.48 mmol g^−1^ (7.1% of CO_2_ adsorption capacities). Therefore, the PC–800 with even minor nitrogen doping, is still a potential selective adsorbent for CO_2_ and N_2_ separation. Figure 8d shows the CO_2_ adsorption capacities at 0 °C for the PC–800 from 0 to 1000 mbar with seven repeated runs. Clearly, the adsorption amounts for the PC-800 keep almost unchanged at 0 °C in the measured pressure ranges within seven runs, demonstrating its high recyclable stability for capture of CO_2_.

## Conclusions

In summary, hetero-oxygen and nitrogen doped porous carbons have been produced via hydrothermal treatment of stem bark with complex multilayered structure from BP as the biomass precursor in diluted sulfuric acid, and following thermal activation by KOH at 800 °C. The as-synthesized hetero-atom porous carbon possesses a partial graphitic structure with a very high specific surface area of 1759 m^2^ g^−1^ and a high pore volume of 0.92 cm^3^ g^−1^. The hetero-atom doped PCs based electrode for supercapacitor displays a high specific capacitance of 416 F g^−1^ at 0.5 A g^−1^, together with superior rate capability and cycling stability. As a solid-state adsorbent, the hetero-atom doped PCs has an excellent CO_2_ adsorption capacity at ambient pressures of up to 6.71 and 4.45 mmol g^−1^ at 0 and 25 °C, respectively. Considering the worldwide abundance and recyclability of BP, its stem bark can be acted as a novel biomass source for large-scale production of the PCs for high-performance supercapacitors and CO_2_ adsorbent.

## Experimental

### Materials

BP stem bark was collected from a local plantation and was prior washed, cut into pieces (1 cm × 1 cm) and dried. All of the other chemicals were analytical grade and were purchased from Aladdin Reagent (Shanghai) Co., Ltd. without further treatment.

### Preparation of Nitrogen Doped Porous Carbon

Nitrogen doped porous carbon were prepared by carbonization and activation of the hydrothermal product of BP stem bark. Detailed procedures are as follows: 10.0 g of BP stem bark and 200 mL of 1 M diluted sulfuric acid were placed in a 250 mL stainless steel autoclave. The autoclave was sealed and heated at 170 °C for 10 h and then allowed to cool to room temperature. The resulting carbonaceous solid, denoted as biochar, was recovered by filtration, washed with distilled water, and dried. The biochar material was chemically activated using potassium hydroxide. The biochar and KOH were thoroughly ground in an agate mortar in a 1:2 mass ratio, and then the mixture was heated at 700 ~ 900 °C (5 °C min^−1^) for 1 h under argon flow. After that, the activated samples were thoroughly washed with 10 wt % HCl and distilled water. Finally, the carbons were dried in an oven at 100 °C for 12 h.

### Structural Characterization

The morphology of the obtained porous carbons was characterized by scanning electron microscopy (SEM, JEOL JSM-6610LV and JEOL S-4800) operated at an acceleration voltage of 10 kV. Transmission electron microscopy (TEM) images were obtained using a JEOL JEM-1011 microscope operating at 200 kV. High-resolution TEM (HRTEM) was performed using a JEM-2100 F microscope operating at an accelerating voltage of 200 kV. The crystallographic information of porous carbons was investigated by powder X-ray diffraction (XRD, Rigaku D/Max 2500PC). Raman spectra were collected on a Renishaw inVia Raman spectrometer. X-ray photoelectron spectroscopy (XPS) was performed on a 1063 photoelectron spectrometer (Thermo Fisher Scientific, England) with Al-Kα X-ray radiation as the X-ray source for excitation. The textural properties were characterized by N_2_ sorption measurements at 77.3 K (Micromeritics TriStar II 3020). The specific surface area was obtained by Brunauer-Emmett-Teller (BET) method. The pore size distribution (PSD) was calculated by the nonlocal density functional theory (NLDFT) method. The total pore volume (*V*_total_) was estimated from the adsorbed amount at a relative pressure *p/p*° of 0.99. Micropore volume (*V*_mic_) was calculated using the t-plot method.

### Electrode Preparation and Electrochemical Measurements

The working electrodes were typically fabricated by mixing the porous carbon as the active material (80 wt%), acetylene black (62 m^2^/g, 10 wt%), and polytetrafluoroethylene (PTFE, 10 wt%) in ethanol and then coated onto the nickel foam current collectors (1 cm × 1 cm) with a spatula. The premade electrodes were pressed under a pressure of 10 MPa for 5 min and finally dried at 130 °C for 12 h in an oven. The symmetric two-electrode supercapacitor was assembled into a total of 2032 stainless steel coin cells in air with nearly identical (both weight and size) electrodes, the electrolyte (6 M KOH) and a glassy fibrous separator.

The capacitive performance of single electrode was studied on a CHI760D electrochemical workstation (CH Instruments Inc., Shanghai, China) using a standard three-electrode system with platinum wire and Hg/HgO electrode as counter and reference electrodes, respectively, in 6 M KOH electrolyte at 25 °C. Cyclic voltammetry (CV), galvanostatic charge-discharge (GCD) technique and alternating current impedance were employed in the electrochemical investigations. CV tests of individual electrode were carried out between −1.0 and 0 V (*vs.* Hg/HgO). GC tests were performed at different current density varying from 0.5 to 50 A g^−1^ in the same potential range as the CV test. The specific capacitance based on GC was calculated using the equation^17^,^18^: *C*_g_ = *I*/(*mdV/dt*), where *I* is the constant current and *m* the mass of active materials, and *dV/dt* is calculated from the slope obtained by fitting a straight line to the discharge curve from the end of the voltage drop to the end of the discharge process. Gravimetric capacitance from GC of the two two-electrode configuration was calculated by using the formula *C*_g_ = 4*I*/(*mdV/dt*).

### CO_2_ Capture Measurements

The CO_2_ adsorption isotherms of the samples were measured using a Micromeritics TriStar II 3020 static volumetric analyzer at 0 and 25 °C. The N_2_ adsorption isotherms of the BP–800 were measured using a Micromeritics TriStar II 3020 static volumetric analyzer at 0 °C. Prior to each adsorption experiment, the sample was degassed for 10 h at 150 °C to ensure that the residual pressure was below 1 × 10^−3^ mbar. After the samples were cooled down to 0 or 25 °C, CO_2_ was introduced into the system. The CO_2_ adsorption capacity in terms of the adsorbed volume under standard temperature and pressure was then recorded. The recycling adsorption test of CO_2_ was performed with a simple regeneration by evacuating at 150 °C for 10 h under a pressure of 1 × 10^−3^ mbar^19^.

## Additional Information

**How to cite this article**: Wei, T. *et al.* A Facile and Low-Cost Route to Heteroatom Doped Porous Carbon Derived from Broussonetia Papyrifera Bark with Excellent Supercapacitance and CO_2_ Capture Performance. *Sci. Rep.* **6**, 22646; doi: 10.1038/srep22646 (2016).

## Supplementary Material

Supplementary Information

## Figures and Tables

**Table 1:** Textural electrochemical and CO_2_ adsorption properties of the PCs.

Sample>	BET SSA (m>^2>^ g>^−1>^)				Pore volume (cm>^3>^ g>^−1>^)				Pore width (nm)>	*C>*_g>_c> (F g>^−1>^)	CO>_2>_d> Adsorption (mmol g>^-1>^)
	Total>	Micro>	External>	Ratio>a>	Total>	Micro>	External>	Ratio>b>			
PC-700	1393	1282	111	11.55	0.63	0.49	0.14	3.50	3.17	314	3.92
PC-800	1759	1539	220	7.00	0.92	0.60	0.32	1.88	3.11	416	4.45
PC-900	1229	350	879	0.40	0.89	0.15	0.74	0.20	2.79	252	3.76

^a^The micropore area to external area ratio.

^b^The micropore volume to external volume ratio.

^c^The *C*_g_ values calculated from GC curves at a current density of 0.5 A g^−1^, see Fig. 5c.

^d^The CO_2_ adsorption capacities measured under ambient pressure at 25 °C.
