# Supplementary material for: A Facile and Low-Cost Route to Heteroatom Doped Porous Carbon Derived from Broussonetia Papyrifera Bark with Excellent Supercapacitance and CO2 Capture Performance
Source: Sci Rep. 2016 Mar 3;6:22646. doi: 10.1038/srep22646 (PMC4776128; doi:10.1038/srep22646)
Supplement: Supplementary Information [file srep22646-s1.pdf]

Supporting Information

A Facile and Low-Cost Route to Heteroatom Doped Porous Carbon Derived from Broussonetia Papyrifera Bark with Excellent Supercapacitance and CO_2 Capture Performance

Tongye Wei [a], Qi Zhang [a] , Xiaolin Wei [a]*, Yong Gao [b], Huaming Li [b]

Xiangtan University, Xiangtan 411105, Hunan Province, P. R. China.

[a] Hunan Key Laboratory of Micro-Nano Energy Materials and Devices, Department of Physics,

[b] College of Chemistry.

*Corresponding author. Tel.: +86 731 58293829; Fax: +86 731 58292061.

E-mail address: xlw@xtu.edu.cn

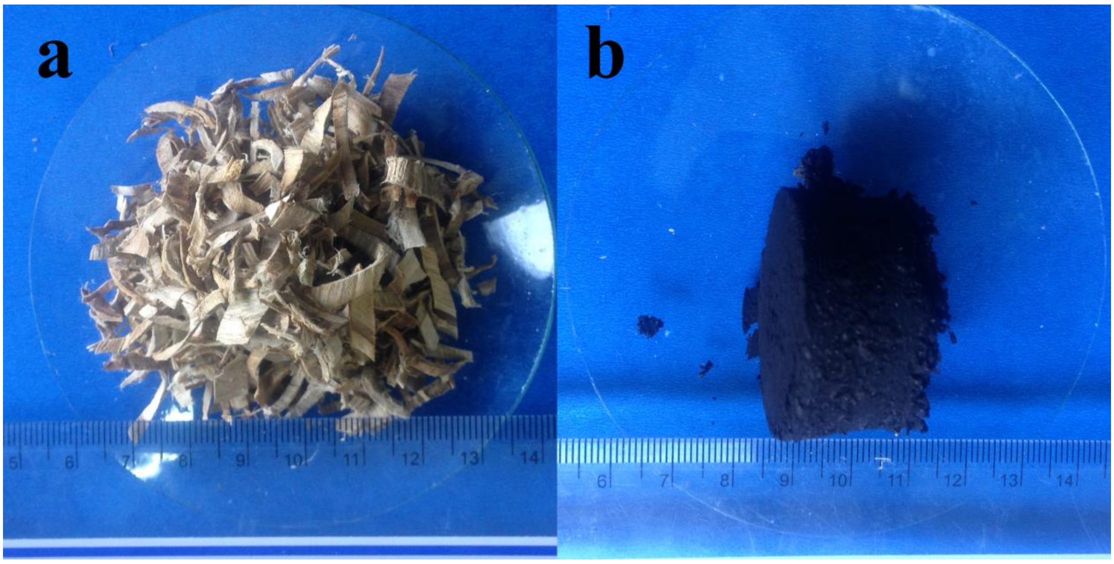

Figure S1. Photographic images of BP bark fiber (a) before and (b) after hydrothermal carbonization.

S1. Material

BP stem bark was collected from a local plantation in china and was prior washed, cut into pieces and dried. All of the other chemicals were analytical grade and were purchased from Aladdin Reagent (Shanghai) Co., Ltd. without further treatment.

S2. Effect of KOH mass radio on the BET Area and Capacitance of the Resultant PCs

Firstly, 10 g BP stem bark was added into 200 mL of 2.0 M H_2SO_4 solution. After stirring at room temperature for 30 min, the mixture was sealed into a Teflon-lined stainless steel autoclave (250 mL) followed by hydrothermal treatment at 170 ℃ for 10 h. The resulting carbonaceous solid, denoted as biochar was collected by filtration washed with distilled water

and dried at 100 ℃ for 24 h. The biochar material was chemically activated using KOH The biochar and KOH were thoroughly ground in an agate mortar in 1:1- 1:3 mass ratio, and then the mixture was heated at 800 ℃(4 ℃ min^{-1}) for 2 h under N_2 flow. The products were denoted as BPC-700-***R***, standing for porous carbons prepared from BP stem bark, where R (R = 1, 2, or 3) stands for the biochar and KOH mass ratio. Figure S2 shows the N2 adsorption-desorption isotherms (a), pore size distributions (b), CV curves tested at a scan rate of 5 mV s^{-1} (c), and GC curves tested at a current density of 0.5 A g^{-1} (d) for the PC-800-1, PC-800-2 and PC-800-3.

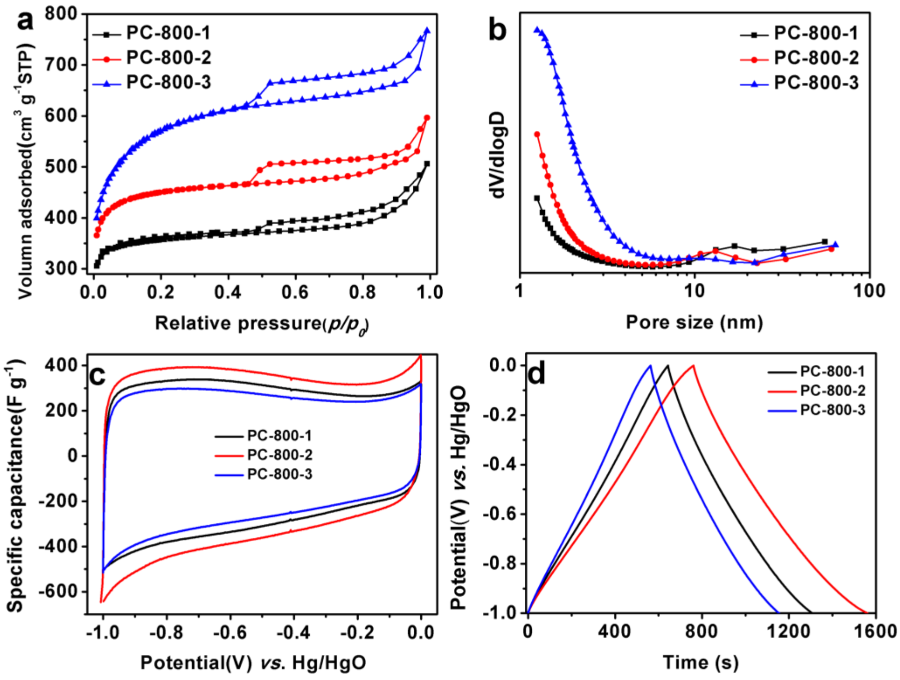

Figure S2. N_2 adsorption-desorption isotherms (a), pore size distributions (b), CV curves tested at a scan rate of 5 mV s^{-1} (c), and GC curves tested at a current density of 0.5 A g^{-1} (d) for the PC-800-*R*.

Table S1. Textural and Electrochemical Properties of PC-800-*R*.

Sample	BET SSA (m^2 g^{-1})				Pore volume (cm^3 g^{-1})				Pore width (nm)	C_g[c] ($F\ g^{-1}$)
	Total	Micro	External	Ratio[a]	Total	Micro	External	Ratio[b]		
CP-800-1	1419	1287	132	9.75	0.85	0.49	0.36	1.36	5.22	327
CP-800-2	1759	1539	220	6.90	0.92	0.60	0.32	1.87	3.11	416
CP-800-3	2135	1384	752	1.84	1.19	0.55	0.64	0.86	2.4	294

[a] The micropore area to external area ratio.

[b] The micropore volume to external volume ratio.

[c] The Cg values calculated from GC curves at a current density of 0.5 A g^{-1}, see Fig. S2d.

S3. Effect of hydrothermal condition on BET Area and Capacitance of Resultant PCs

Firstly, 10 g BP stem bark was added into 200 mL of 1.0-3.0 M H_2SO_4 solution. After stirring at room temperature for 30 min, the mixture was sealed into a Teflon-lined stainless steel autoclave (250 mL) followed by hydrothermal treatment at 140-200 ℃ for 10 h. The resulting carbonaceous solid, denoted as biochar was collected by filtration washed with distilled water and dried at 100 ℃ for 24 h. The biochar material was chemically activated using KOH The biochar and KOH were thoroughly ground in an agate mortar in 1:2 mass ratio, and then the mixture was heated at 800 ℃ (4 ℃ min^{-1}) for 2 h under N_2 flow. The products were denoted as PC-700-***H***, standing for porous carbons prepared from BP stem bark, where ***H*** (H = 1M , 3M or 140 ℃, 200 ℃) stands for the hydrothermal condition. (For example PC-800-1M stand

for 1M H_2SO_4, hydrothermal temperature 170 ℃, whereas 140 ℃ stand for 2M H_2SO_4, hydrothermal temperature 140 ℃) Figure S3 shows the N_2 adsorption-desorption isotherms (a), pore size distributions (b), CV curves tested at a scan rate of 5 mV s^{-1} (c), and GC curves tested at a current density of 0.5 A g^{-1} (d) for the PC-800-1M, PC-800-3M, PC-800-140 ℃ and PC-800-200 ℃.

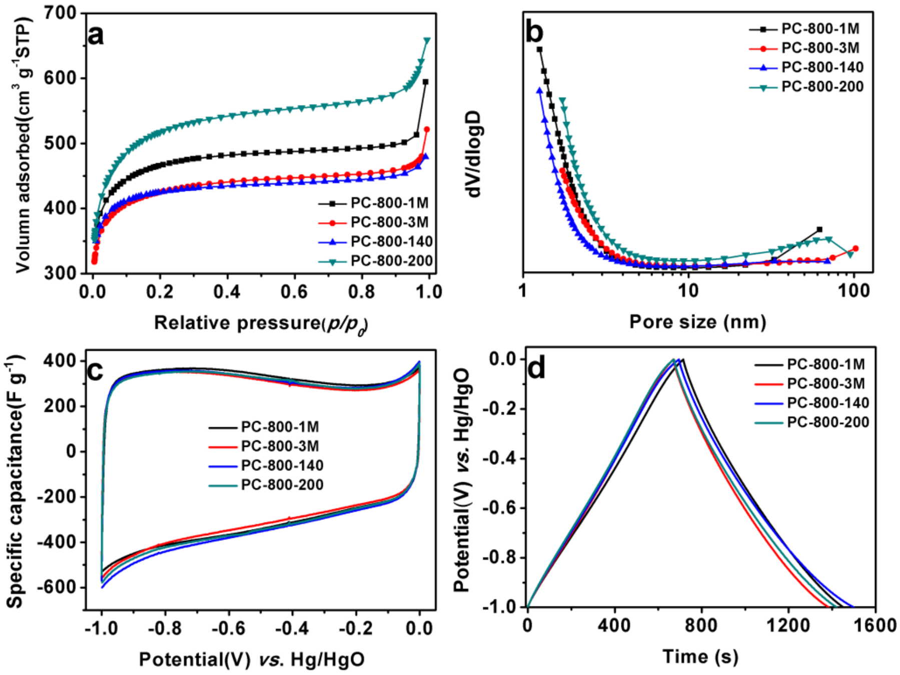

Figure S3. N_2 adsorption-desorption isotherms (a), pore size distributions (b), CV curves tested at a scan rate of 5 mV s^{-1} (c), and GC curves tested at a current density of 0.5 A g^{-1} (d) for the PC-800-*H*.

Table S2. Textural and Electrochemical Properties of PC-800-*H*.

Sample	BET SSA ($m^2 g^{-1}$)				Pore volume ($cm^3 g^{-1}$)				Pore width (nm)	C_g[c] ($F g^{-1}$)
	Total	Micro	External	Ratio[a]	Total	Micro	External	Ratio[b]		
CP-800-140	1612	1292	320	4.04	0.81	0.52	0.29	1.79	4.59	410
CP-800-200	1965	1491	474	3.14	1.02	0.59	0.43	1.37	4.28	393
CP-800-1M	1767	1458	309	4.71	0.92	0.59	0.33	1.79	2.65	388
CP-800-3M	1665	1455	210	6.92	0.74	0.57	0.17	3.35	2.13	376

[a] The micropore area to external area ratio.

[b] The micropore volume to external volume ratio.

[c] The Cg values calculated from GC curves at a current density of 0.5 A g^{-1}, see Fig. S3d.

Table S3. C and N Contents Evaluated from XPS.

Samples	XPS (at.%)			Percentage (at.%)		
	C	N	O	pyridinic-N	pyrrolic-N	quaternary-N
PC-700	87.48	1.61	10.91	27.5	39.9	32.6
PC-800	89.48	1.43	9.09	11.2	50.4	38.4
PC-900	92.59	0.99	6.42	26.4	24.3	49.3
